# Shellfish Face Uncertain Future in High CO_2_ World: Influence of Acidification on Oyster Larvae Calcification and Growth in Estuaries

**DOI:** 10.1371/journal.pone.0005661

**Published:** 2009-05-27

**Authors:** A. Whitman Miller, Amanda C. Reynolds, Cristina Sobrino, Gerhardt F. Riedel

**Affiliations:** 1 Smithsonian Environmental Research Center, Edgewater, Maryland, United States of America; 2 Universidad de Vigo, Vigo, Pontevedra, Spain; Mt. Alison University, Canada

## Abstract

**Background:**

Human activities have increased atmospheric concentrations of carbon dioxide by 36% during the past 200 years. One third of all anthropogenic CO_2_ has been absorbed by the oceans, reducing pH by about 0.1 of a unit and significantly altering their carbonate chemistry. There is widespread concern that these changes are altering marine habitats severely, but little or no attention has been given to the biota of estuarine and coastal settings, ecosystems that are less pH buffered because of naturally reduced alkalinity.

**Methodology/Principal Findings:**

To address CO_2_-induced changes to estuarine calcification, veliger larvae of two oyster species, the Eastern oyster *(Crassostrea virginica),* and the Suminoe oyster (*Crassostrea ariakensis)* were grown in estuarine water under four *p*CO_2_ regimes, 280, 380, 560 and 800 µatm, to simulate atmospheric conditions in the pre-industrial era, present, and projected future concentrations in 50 and 100 years respectively. CO_2_ manipulations were made using an automated negative feedback control system that allowed continuous and precise control over the *p*CO_2_ in experimental aquaria. Larval growth was measured using image analysis, and calcification was measured by chemical analysis of calcium in their shells. *C. virginica* experienced a 16% decrease in shell area and a 42% reduction in calcium content when pre-industrial and end of 21^st^ century *p*CO_2_ treatments were compared. *C. ariakensis* showed no change to either growth or calcification. Both species demonstrated net calcification and growth, even when aragonite was undersaturated, a result that runs counter to previous expectations for invertebrate larvae that produce aragonite shells.

**Conclusions and Significance:**

Our results suggest that temperate estuarine and coastal ecosystems are vulnerable to the expected changes in water chemistry due to elevated atmospheric CO_2_ and that biological responses to acidification, especially calcifying biota, will be species-specific and therefore much more variable and complex than reported previously.

## Introduction

During the past 200 years the combustion of fossil fuels, deforestation, and land development have increased atmospheric concentrations of carbon dioxide by 36% and the rate of CO_2_ emission is expected to increase during the coming century [1]–[3]. Approximately one third of all anthropogenic CO_2_ has been absorbed by Earth's oceans [4]–[5]. Although the ocean has partially absorbed recently liberated anthropogenic atmospheric CO_2_, this has come at the expense of significantly reduced pH (acidification) and altered carbonate chemistry in the ocean's surface waters. Since ca. 1760, the pH of the ocean's surface (the upper 200 m of the water column) has decreased by approximately 0.1 of a unit, and further reductions of 0.1 to 0.5 units are expected during the next 100 years [6]–[8]. There is widespread concern that these changes will produce irreversible ecological regime shifts in marine habitats, such as massive reductions in coral reef habitats and their associated biodiversity as well as reduced availability of carbonate ions for calcifying biota [9]–[11].

To date most research has focused on CO_2_-induced acidification in fully marine waters, while little attention has been devoted to lower salinity estuaries and temperate nearshore ecosystems. But coastal and estuarine biomes are among the most biologically productive and maintain some of the most extensive and measurable ecosystem services (e.g., commercial and recreational fisheries, fish and invertebrate nursery grounds, water purification, flood and storm surge protection, human recreation). Because they are shallower, less saline, and have lower alkalinity [12], estuaries and coastal marine habitats are more susceptible to changes in pH than the open ocean. Estuaries are also susceptible to substantial enrichment in CO_2_, produced by the respiration of both natural and anthropogenic carbon. While many estuaries already have high and variable pCO_2_ [13], atmospheric CO_2_ enrichment will shift the baseline toward even higher values. For these reasons, these ecosystems will likely experience more acute impacts from elevated CO_2_ in coming decades.

A direct consequence of CO_2_-induced acidification is the reduction of carbonate ion concentration [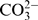] in the water column. Of special concern in marine and estuarine systems is the effect of rising CO_2_ on the saturation state of water with respect to calcium carbonate (Ω), where
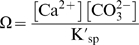
and K′_sp_ is the apparent solubility product [14]–[15]:
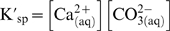
Ω is therefore a proxy for the ease with which organisms can produce calcium carbonate (CaCO_3_). The water's saturation states with respect to aragonite and calcite, the two most commonly biomineralized forms of CaCO_3_, are diminished with increased partial pressures of CO_2_ (*p*CO_2_) [15].

In estuaries, Ω naturally decreases with decreasing salinity due to gradients in pH, Ca^2+^ and 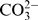 produced by the dilution of seawater with river water (see Cai and Wang [13]). A recent study by Salisbury et al. [16] indicates that seasonal discharges of acidic riverine water will further exacerbate acidification in estuaries, suggesting the possibility of negative impacts to shell fisheries.

**Figure 1:**
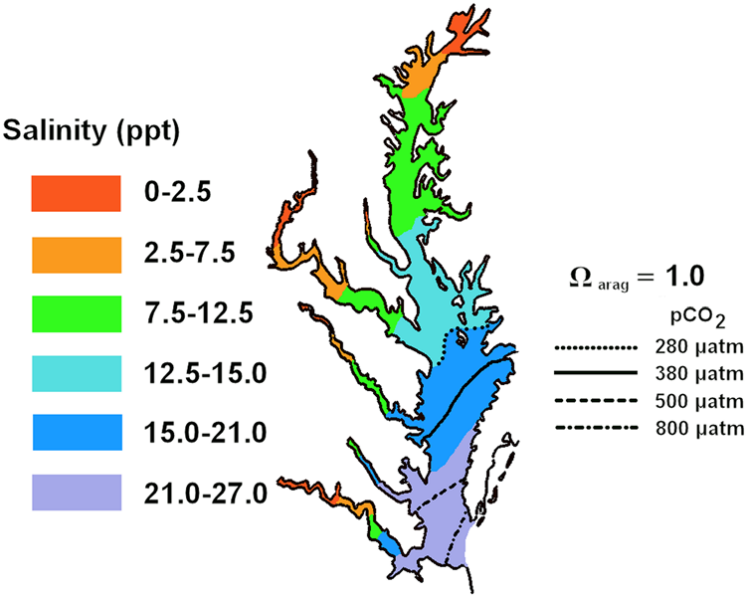
Projected mean summer positions of aragonite compensation points (Ω_arag_ = 1.0) for Chesapeake Bay. Theoretical aragonite compensation positions were plotted under past, present and predicted atmospheric CO_2_. Aragonite saturation was calculated using the program CO_2_SYS.XLS from alkalinity and atmospheric *p*CO_2_ at 25°C and 0 mbar pressure, assuming conservative mixing of Atlantic and the Susquehanna River alkalinities, and mean summer salinities (1985–2006 – data from Chesapeake Bay Program [47]). Aragonite is supersaturated seaward of compensation lines.

In Chesapeake Bay, Wong [12] showed that water from the James River and ocean mix conservatively and that salinity and total alkalinity (TA) are linearly related from approximately 5 psu to 32 psu. At ∼18 psu, TA in the Bay is ∼1250 µmol/kg-SW. If conservative mixing of water and equilibrium with current atmospheric CO_2_ (380 µatm) are assumed, the aragonite compensation point (i.e., Ω_arag_ = 1.0) at summer temperatures should lie near the 18 psu isopleth. As atmospheric CO_2_ increases, assuming Ca^2+^ and alkalinity remain at present levels, the aragonite compensation point in estuaries will shift seaward toward higher salinities. We contend that the aragonite compensation point has already shifted significantly toward the Bay's mouth since the beginning of the industrial era (Fig. 1). The compensation point for calcite, which is approximately 1.5 times less soluble than aragonite [14] occurs at lower salinities and will shift seaward in a similar way. In reality, estuaries are frequently not in equilibrium with the atmosphere, however, a change in atmospheric *p*CO_2_ will shift the baseline equilibrium to which estuaries tend, and result in wider spread elevated *p*CO_2_.

We expect bivalve larvae to be more vulnerable than adults to increased CO_2_ because larvae biomineralize aragonite, the more soluble form of CaCO_3_, rather than calcite, the predominant material used in adult shell. Weiss et al. [17] further indicate that during biomineralization some bivalve larvae, (e.g., *Crassostrea gigas* and *Mercenaria mercenaria*) generate an even more soluble amorphous CaCO_3_ as an ephemeral precursor to crystalline aragonite, and because larvae are generally less robust than adults to a variety of stresses. If larvae are indeed more susceptible to acidification, it may lead to their reduced performance, or even failure, ultimately leading to negative effects on oysters and other shellfish populations. Kurihara et al. observed at very high *p*CO_2_ (2268 µatm) that embryonic development and shell formation in *Crassostrea gigas* was inhibited during the initial 48 h following fertilization [18]. How shifting carbonate chemistry will alter the ecological structure and function in benthic communities remains a critical gap in our knowledge [1]–[2].

We conducted experiments on the veliger larvae of two closely related euryhaline oyster species, the Eastern oyster (*Crassostrea virginica* [Gmelin 1791]) and the Suminoe oyster (*Crassostrea ariakensis* [Fujita 1913]), under conditions of continuously controlled pH/pCO_2_ for up to 28 days. *C. virginica* is native to the Western Atlantic, but the recent landings are just 1% of their historical maximum in Chesapeake Bay, due in large measure to overfishing and disease [19]–[20]. The introduction of *C. ariakensis* to Chesapeake Bay has been considered in the hopes of restoring the oyster fishery and important ecosystem functions, such as water filtration and reduction of nutrients and sediment in the water column [21]. We hypothesize that (a) oyster larvae will exhibit reduced growth and calcification at elevated *p*CO_2_ and (b) larval development will be adversely and measurably affected when Ω_arag_<1.0. By extension, oyster fisheries, other shellfish, and calcifying benthic fauna are expected to be negatively influenced by acidification.

## Materials and Methods

All oyster larvae tested were obtained from the Virginia Institute for Marine Science's Eastern Shore Laboratory, Wachapreague, VA, where *C. virginica* and *C. ariakensis* were spawned in quarantined facilities. Experiment *Ca* 1 larvae were derived from *C. ariakensis* stocks originating in the Ariake Sea of Japan but accidentally transferred to the west coast of the US with shipments of *C. gigas* in the 1970s [22]. *Cv* 1 were derived from Wachapreague seaside *C. virginica* stocks. Table 1 summarizes all experimental parameters for experiments *Cv* 1 and *Ca* 1. At 72 hours post fertilization, D-stage larvae were transferred from Wachapreague to a quarantined laboratory at the Smithsonian Environmental Research Center in Edgewater, MD, and placed into culture. To test the effects of elevated *p*CO_2_, reduced pH, and aragonite saturation state on larval growth and calcification, we cultured oyster larvae under controlled conditions that simulated a typical summer estuarine environment in upper mesohaline reaches of the Chesapeake Bay: salinity = 18 psu, temperature = 25°C, day/night cycle = 14:10 hrs. Experimental treatments were blocked across two incubators (Percival I-36 Controlled Environment Chambers with Philips 700 series 32 W fluorescent bulbs) and individual aquaria randomly positioned within each incubator. Incubator A contained 280 µatm and 380 µatm *p*CO_2_ treatments and incubator B contained 560 µatm and 800 µatm for all experiments. Prior to experiments, the PAR irradiance was measured inside each incubator and shown to be similar (A = 167.7±5.3 µE m^−2^ s^−1^ compared with B = 169.2±7.3 µE m^−2^ s^−1^ (mean±SEM).

**Table 1:** Carbonate chemistry parameter values/sources and culture conditions for Eastern oyster *(Crassostrea virginica)* and Suminoe oyster *(C. ariakensis)* larvae experiments.

Parameter	*C. virginica (Cv 1)*				*C. ariakensis (Ca 1)*				Parameter Source
Simulation date	Pre-IR	2008	2050	2100	Pre-IR	2008	2050	2100	
Target pCO_2_ (µatm)	280	380	560	800	280	380	560	800	
Mean pCO_2_ (µatm)	284	389	572	840	291	386	581	823	CO_2_SYS calc.
SEM	4.8	7.9	10.8	17.4	3.8	6.7	13.6	11.8	
Mean hourly pH	8.16	8.06	7.91	7.76	8.17	8.08	7.92	7.79	Direct Measure
SEM	0.002	0.005	0.005	0.006	0.004	0.006	0.008	0.006	
Mean TA (µmol/kg)	1229	1268	1283	1289	1297	1324	1357	1360	CO_2_SYS calc.
SEM	15.7	18.4	17.2	15.7	16.6	15.5	23.9	16.4	
Mean TDIC (µmol/kg)	1126	1188	1232	1265	1187	1237	1301	1331	Direct Measure
SEM	15.1	17.8	16.9	15.7	15.3	14.6	23	15.8	
Mean [CO_2_] (µmol/kg)	8.8	12	17.7	25.9	9	11.9	18	25.4	CO_2_SYS calc.
SEM	0.15	0.24	0.33	0.54	0.12	0.21	0.42	0.36	
Mean [HCO_3_^−^] (µmol/kg)	1045	1116	1169	1206	1100	1159	1234	1269	CO_2_SYS calc.
SEM	14.1	16.8	16	14.9	13.9	13.6	21.7	15	
Mean [CO_3_^−^] (µmol/kg)	72.4	60.4	45.1	32.7	78.3	65.7	49.6	36.9	CO_2_SYS calc.
SEM	0.8	1	0.7	0.6	1.4	1.3	1.4	0.8	
Mean Ω_arag_	1.2	1	0.8	0.6	1.3	1.1	0.8	0.6	CO_2_SYS calc.
SEM	0.01	0.02	0.01	0.01	0.02	0.02	0.02	0.01	
Salinity (psu)	18.2	18.2	18.2	18.2	18.2	18.2	18.2	18.2	Direct Measure
SEM	0.04	0.04	0.04	0.04	0.03	0.03	0.03	0.03	
Duration (d)	26	26	26	26	28	28	28	28	

Larvae were grown under four *p*CO_2_ conditions (280, 380, 560, 800 µatm), simulating equilibration with atmospheric CO_2_ in the preindustrial era, present (2008), 2050 and 2100 CE. All experiments were conducted at 25°C and under a 14 h:10 h light:dark cycle, simulating summer growing conditions in Chesapeake Bay. TA = Total Alkalinity,TDIC = Total Dissolved Inorganic Carbon.

Natural sea water was collected from Sinepuxent Bay, MD (∼28 psu) and Delaware Bay, DE (∼24 psu) and filtered through a 0.2 µm filter and diluted with deionized water to 18 psu. An open cell, two-point titration [23] determined the TA of the water just prior to the onset of each experiment. Total alkalinity and *p*CO_2_ targets for pre-industrial (*p*CO_2_ = 280 µatm), present day (*p*CO_2_ = 380 µatm), year 2050 (*p*CO_2_ = 560 µatm) and year 2100 (*p*CO_2_ = 800 µatm) settings, as projected by the IS92a “business as usual” scenario [24] were then entered into the computer program CO_2_SYS.XLS [25]. The program was parameterized specifically with the two dissociation constants K_1_ and K_2_ for carbonic acid in estuarine waters of Cai and Wang [13] to determine corresponding experimental pH levels.

Larvae were grown in 4-L polycarbonate aquaria, under four *p*CO_2_ treatments, and each treatment included three replicate aquaria that were independently controlled for *p*CO_2_/pH (n = 12). Filtered water samples (0.2 µm) were collected from each aquarium every few days throughout the experiments for total dissolved inorganic carbon (TDIC) analysis. (To maintain water quality, water was changed in all aquaria every two days. TDIC samples were taken 24–48 hours after a water change.) Water samples were capped without head space and analyzed immediately, or stored at 4°C and analyzed within 48 hrs. TDIC was measured using a total organic carbon analyzer, outfitted with a phosphoric acid inorganic carbon reagent reaction chamber and non-dispersive infrared detector (Schimadzu TOC-V with IC reactor kit). TDIC was then partitioned into *pCO_2_*, bicarbonate, and carbonate and TA calculated with CO2SYS.XLS [25] (Table 1.) Experiments were ended when larvae of the largest size classes achieved competence as indicated by the presence of eye spots.

Experiments *Cv* 1 (*Crassostrea virginica*) and *Ca* 1 (*C. ariakensis*) were inoculated with 15,000 four day-old D-stage larvae respectively, and were grown for up to 28 days. Aquaria received controlled quantities of the microalgae *Isochrysis galbana* (Prymnesiophyceae) as food. Microalgae were grown in semi-continuous cultures using f/2-Guillard medium. Equal quantities of microalgae were pipetted daily from cultures of known density into each aquarium, with daily doses increasing as larvae grew larger. Target inoculation densities in each aquarium increased from 1.5·10^4^cells·ml^−1^ at the beginning of an experiment to 1.0·10^5^cells·ml^−1^ during the finals days, when larvae were larger and ingesting more food.

The *p*CO_2_ in each aquarium was sustained by intermittent bubbling with CO_2_-enriched air (commercially available, certified 1.0% CO_2_) and continuous aeration with CO_2_-stripped air. Soda-lime filters were used to strip CO_2_. Continuous aeration with CO_2_-stripped air oxygenated and mixed the water, while simultaneously driving CO_2_ out of the water and pushing pH upward. Each aquarium was continuously monitored with a pH probe (NBS scale) and control system. Once a target pH set point was exceeded, an automated controller opened a solenoid and delivered the 1% CO_2_ air mixture to an aquarium, thereby driving pH downward. The flow of CO_2_ was automatically stopped when the pH set point was reached. Based on hourly pH measurements, the negative feedback control system was shown to maintain pH (and *p*CO_2_) at near constant levels for up to 4 weeks. The four pH set points used corresponded to desired target *p*CO_2_ treatments for water of known total alkalinity. Probes were calibrated prior to the experiment using NIST traceable buffers (pH = 4.00 and 7.00), recalibrated at least one time during the experiment, and again at the conclusion of the experiment to ensure proper operation.

At the end of each experiment, larvae from each aquarium were collected and fixed in 95% ethanol. Random samples were taken from each replicate and photographed at 20× magnification; (n = 205±6.4 shells per replicate (mean±SEM)). Larval shells were positioned on their sides and photographed in silhouette. Outer shell areas were measured using digital image analysis [26] (Scion Image, ver. 4.0.3.2). All areas are reported as total outer surface areas (i.e., 2× photographed area = total surface area of both valves). The same samples were rinsed with deionized water to remove sea salts and ethanol, and the shells dissolved in trace metal grade HCl, and diluted to a known volume. Inductively coupled plasma/optical emission spectroscopy (ICP/OES) was used to measure the mean calcium content per shell from each replicate sample across treatments.

For each experimental treatment, the mean mass of CaCO_3_ per unit area (µg/µm^2^) was determined by dividing the mean CaCO_3_ content of shells by the mean shell area. These values were then divided by the density of crystalline aragonite (2.93 g/cm^3^) [27] to estimate the mean apparent aragonite thickness of larval shells. However, this uniformly overestimates shell thickness slightly, because image analysis calculated shell area in silhouette, thereby underestimating the true three-dimensional shell surface. This bias should not affect comparisons with treatments for the same organism.

## Results

### Larval Development

Larval development proceeded from 96 h post fertilization to the eyed veliger and pediveliger stages (i.e., achieved competence) among the largest size classes in all treatments and across all experiments. Experiments *Cv* 1 and *Ca* 1 ran to larval ages of 30 d and 32 d, respectively.

### Shell Area

**Figure 2:**
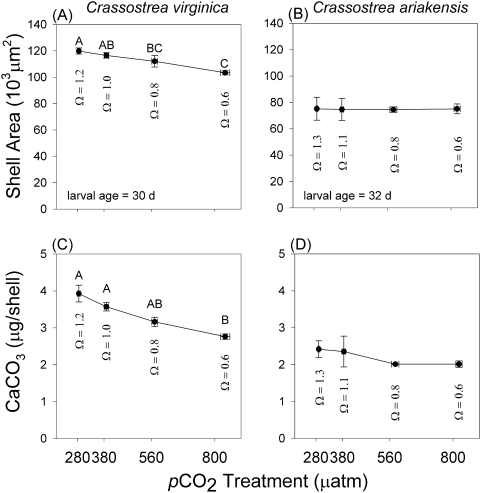
Effects of *p*CO_2_ treatment on larval shell growth and calcification. Mean shell areas±SEM (µm^2^) (panels A and B) and mean shell CaCO_3_ content±SEM (µg/shell) (panels C and D) reported by *p*CO_2_ treatment±SEM (µatm) for two oyster species. Corresponding aragonite saturation states (Ω_arag_) are indicated for each treatment. Statistical differences determined by ANOVA and Tukey HSD tests. *C. virginica* grew more quickly than *C. ariakensis* under all treatments but experienced reduced growth at high *p*CO_2_. The growth of *C. ariakensis* was not noticeably affected by elevated *p*CO_2_ or aragonite saturation. *C. virginica* calcified less at elevated *p*CO_2_ and Ω_arag_<1.0 (panel C), whereas calcification of *C. ariakensis* was not significantly influenced by elevated *p*CO_2_ or aragonite saturation (panel D).

**Figure 3:**
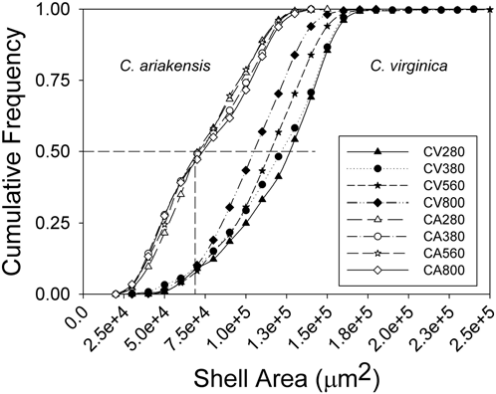
Effects of *p*CO_2_ treatment on cumulative size frequency of larval shells (µm^2^/shell) for two oyster species. Black symbols and curves on right show results from *Crassostrea virginica*, experiment *Cv 1*, age = 30 d. Open symbols and left curves plot *C. ariakensis* results (experiment *Ca 1*, age = 32 d). *PCO_2_ treatments = 280, 380, 560, 800* µatm. Number of shells per treatment = 615±26.7 (mean±SEM). *C. virginica* grew to markedly larger sizes than *C. ariakensis*. Ninety percent of *C. virginica* shells were as large as or larger than the median *C. ariakensis* size class (dashed line). Growth of *C. virginica* was influenced by *p*CO_2_ treatment and *C. ariakensis* was unaffected.

*Crassostrea virginica* grew more slowly at elevated *p*CO_2_ than at either 280 or 380 µatm treatments, as indicated by analysis of variance (*Cv* 1: *F_3,8_* = 6.605, *n* = 12, *P* = 0.015, Fig. 2a). No significant differences in shell growth were observed across *p*CO_2_ treatments for *C. ariakensis* (*Ca* 1: *F_3,8_* = 0.024, *n* = 12, *P* = 0.995, Fig. 2b). Cumulative shell size frequencies for each species and treatment reveal: (1) that ninety percent of *C. virginica* shells exceeded the median size class of *C. ariakensis* and (2) clear treatment effects in *C. virginica* growth but none in *C. ariakensis* (Fig. 3).

### Calcification

The amount of CaCO_3_ biomineralized by *Crassostrea virginica* larvae decreased as *p*CO_2_ increased (Fig. 2c). Analysis of variance confirmed significant differences among *p*CO_2_ treatments (*Cv* 1: *F_3,8_* = 11.8026, *n* = 12, *P* = 0.0026). By contrast, no clear trends or differences in biomineralization were apparent for *C. ariakensis* (*Ca* 1: *F_3,7_* = 0.6451, *n* = 11, *P* = 0.6103, Fig. 2d).

### Aragonite Saturation

In each experiment, aragonite conditions ranged from supersaturating (Ω_arag_ = 1.2−1.3) to undersaturating (Ω_arag_ = 0.6). *C. ariakensis* was unaffected by differences in aragonite saturation, but growth and calcification in *C. virginica* were significantly curtailed when *p*CO_2_ was high and Ω_arag_<1.0 (Fig. 2). Importantly, both species achieved net growth even when the saturation state of aragonite was well below 1.0.

### Shell Thickness

**Figure 4:**
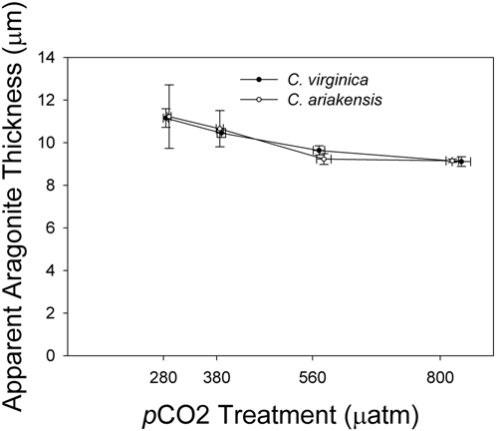
Effects of *p*CO_2_ treatment on apparent aragonite shell thickness for two oyster species. Apparent aragonite thickness (µm) of larval shells = mean CaCO_3_ content (µg)/mean shell area (µm^2^)/aragonite density (2.93×10^−6^ µg/µm^3^). Aragonite thickness was similar across species and varied little across *p*CO_2_ treatments.

Although cross sectional measurements of larval shells were not performed directly, we derived an estimate of average shell thickness that is theoretically attributable to aragonite volume. For each experimental treatment, the mean CaCO_3_ content per shell was divided by the corresponding mean shell area (µg/µm^2^). This value was then divided by the density of crystalline aragonite (2.93×10^−6^µg/µm^3^), leaving the mean apparent aragonite thickness in µm. The mean apparent aragonite thickness was strikingly similar across treatments and experiments (ranging from 9 to 11 µm) regardless of species or larval age (Fig. 4).

A second pair of similar experiments was conducted using a modified design (i.e., fed with a two algal species diet, use of a different strain of *C. ariakensis*, and a greater starting larval density). Because of ciliate contamination in one of the *C. virginica* treatments, the experiments had to be prematurely terminated. Nevertheless, at larval ages of 14 d, results were similar to those reported above, indicating significantly reduced shell area and calcium content for *C. virginica* at elevated *p*CO_2_, but no significant *p*CO_2_ effects for *C. ariakensis*.

## Discussion

Most ocean acidification studies have focused on either warm water corals or pelagic biota [1]–[2], [28]. In general, marine fauna exhibit reductions in calcification at elevated *p*CO_2_ (e.g., scleratinian corals, Gattuso et al. [29], Langdon [9]; pteropods, Feely et al. [15], Orr et al. [8]; foraminifera, Bijima et al. [30]; see Fabry et al. [28] for review of marine faunal responses to elevated *p*CO_2_). As a group, coccolithophores react more variably, appearing to show species-specific responses to elevated CO_2_ [31], with Iglesias-Rodriguez et al. [32] reporting increased calcification in *Emiliania huxleyi* at *p*CO_2_ at 750 µatm. Experiments to date have largely simulated elevated atmospheric CO_2_ in fully marine settings (i.e., high salinity), meaning that water was supersaturated for CaCO_3_ under all challenge treatments due the relatively high total alkalinity of the seawater.

In cases where marine mollusks have been subjected to aragonite undersaturation, the results have generally been deleterious. For example, Kurihara et al. [18] observed that calcification and shell formation was inhibited during early development of *Crassostrea gigas* larvae when seawater was undersaturated with respect to aragonite (Ω_arag_ = 0.68). When adult pteropods (*Clio pyramidata)* were exposed to seawater that was undersaturated for aragonite, their shells began to dissolve within 48 hours [8], [15], [28]. Green et al. [33] showed that newly set juvenile hard shell clams *(Mercenaria mercenaria*, <2.0 mm*)* grown in benthic sediments with porewater undersaturated for CaCO_3_ showed signs of corrosion and significantly increased mortality. Hall-Spencer et al. [34] have observed that the shells of adult gastropods living in ultra high *p*CO_2_ habitats associated with natural underwater volcanic CO_2_ vents in the Mediterranean also dissolve when Ω≪1.0.

In contrast, when *C. virginica* and *C. ariakensis* larvae were cultured continuously from 96 h post fertilization (D-stage) to ∼30 d in typical estuarine conditions (salinity = 18 psu, TA ≈1225 µmol/kg) and exposed to elevated *p*CO_2_ levels, both species appeared to grow, calcify and develop normally with no obvious morphological deformities, despite conditions of significant aragonite undersaturation (Ω_arag_ = 0.6−0.7). These findings demonstrate the physiological capacity of oyster larvae to withstand prolonged exposure (up to 28 days) to high *p*CO_2_ and aragonite undersaturation, and run counter to expectations that aragonite shelled larvae should be especially prone to dissolution at high *p*CO_2_ [28]. It should not be surprising that some mollusks can grow in undersaturated conditions; most fresh water mollusks are clearly well adapted to such conditions. Nevertheless, as atmospheric CO_2_ continues to rise, all calcifying organisms will be encountering conditions that they have not experienced in recent geologic history.

When Carriker and Palmer [35] studied the normal growth and development of *C. virginica* larval shells using scanning electron microscopy, they found uniform, extremely thin shells, 4–6 µm across a range of larvae 100–350 µm in length. The thinness of the shells for their area, ca. 2–4% of the total length, is likely an adaptation to planktonic living. In our study, both oyster species generated larval shells that were of similar mean thickness, regardless of *p*CO_2_, Ω_arag_, or shell area. We interpret the pattern of similar shell thickness as further evidence of normal larval shell development.

Regardless of their shared physiological tolerance to undersaturated aragonite, *C. virginica* and *C. ariakensis* have divergent responses to elevated *p*CO_2_. *C. virginica* demonstrates consistently reduced growth and calcification at high *p*CO_2_. Conversely, *C. ariakensis* larvae are not differentially affected by *p*CO_2_, but do grow and calcify more slowly than *C. virginica* under all *p*CO_2_ treatments. These differences suggest that CO_2_-induced acidification is species-specific and will have unpredictable consequences in estuaries and nearshore ecosystems, even in closely related species.

From strictly thermodynamic considerations, the process of calcification in undersaturated environments (Ω_arag_<1.0) must require the input of exogenous energy to go forward. Weiner and Dove [36] describe two predominant processes of biomineralization widely used by marine and estuarine invertebrates. Extracellular mineralization is used by mollusks, scleratinian corals, bryozoans and some foraminifera and requires active pumping of ions or secretion and active transport of vesicles containing aqueous solutions of high ionic concentrations across cell membranes to produce conditions favorable for calcification. Coccolithophores, echinoderms, and other formaminifera use intracellular biomineralization, whereby nucleation of mineral structures occurs directly inside vesicles using similar active transport systems. The resulting carbonate structures are then secreted across the cell membrane (Weiner and Dove [36] and references therein). As aragonite becomes less saturated, the energetic costs for biomineralization should become progressively more expensive.

We do not perceive aragonite undersaturation as a “formal” barrier to calcification by marine or estuarine organisms in general. Aragonite saturation state is rather a convenient index to the ease with which biomineralization can be carried out. The higher the saturation state, the greater the relative availability of both Ca^2+^ and 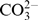, and the lower the energy requirement for biochemical pumping to generate local supersaturation used when producing shell material. Just as species differ according to many environmental requirements, they likely differ in their ability or efficiency to supply energy for calcification. Marine taxa such as corals and pteropods, which are well adapted to marine conditions where both Ca^2+^ and 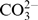 have been abundant and relatively constant for millions of years, may have problems calcifying as 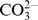 declines due to anthropogenic CO_2_ enrichment, even though their water remains above saturation for both aragonite and calcite. In contrast, larvae of the euryhaline oysters *C. virginica* and *C. ariakensis*, cultured in mesohaline conditions with lower Ca^2+^ and CO_3_^2+^ are able to generate and maintain aragonite shells when aragonite is somewhat below saturation. Although calcification is suppressed in lower saturation states for *C. virginica, C. ariakensis* is less affected by these conditions. Estuaries and open oceans differ substantially in their physical and chemical characteristics and dynamics, as do the evolutionary histories of their biota. For these reasons, expectations about the biological response to increasing atmospheric CO_2_ and acidification ought to be different as well.

Because of extreme spatial and temporal heterogeneity in estuaries and coastal systems, uniform equilibrium with atmospheric CO_2_ is neither expected nor achieved [37]–[38]. Estuarine habitats where shellfish settle and live often experience localized high *p*CO_2_, up to as much as 6000 µatm, due to benthic respiration [39]. Our results suggest that for one key species, *C. virginica*, growth and calcification are diminished at relatively low enhancements of *p*CO_2_, conditions that fall within the bounds of variation that currently exist in its native habitat. When combined with existing natural forcing, future increases to *p*CO_2_ may influence significantly the success of larvae, and ultimately the success and distribution of these oysters in Chesapeake Bay and similar systems.

We predict that as atmospheric CO_2_ concentrations increase, conditions in estuaries will be less favorable for calcification in all salinity zones and will pose new environmental stresses on their inhabitants. For some calcifying species, the added energetic burden of producing shell under less favorable conditions may restrict growth. Our results suggest that this will be the case for *C. virginica*, but not necessarily for *C. ariakensis.* As energy costs mount, calcifying biota may be faced with a trade-off, either to allocate more energy to calcification at the cost of growth and stored energy reserves, or to accept a lesser degree of calcification in exchange for greater growth and more stored energy.

When invertebrate larvae become physiologically stressed from chemical and physical factors encountered in their environment, they sometimes delay settlement and metamorphosis. Pechenik [40] lists temperature, salinity, nutrition, low dissolved oxygen, pollution, and ultraviolet radiation as possible stressors that may induce delayed metamorphosis, and further indicates that larvae are more sensitive than adults to such stressors (Pechenik [41] and references therein). High *p*CO_2_ and reduced CaCO_3_ saturation may produce similar effects. For planktotrophic larvae, even minor increases in energy expenditure for shell production may reduce energy reserves that are critical for larval growth and survival. Metamorphosis is energetically demanding and relies entirely on energy stores accumulated during the larval stage [41]–[42]. If larval growth and calcification are adversely affected by CO_2_-induced acidification, larvae may well be energetically disadvantaged as they attempt metamorphosis, thereby suffering reduced survivorship and fitness. Furthermore, Pechenik [41] indicates that latent larval effects (i.e., impacts to post-metamorphic growth and mortality due to larval experience) may be common and species-specific among marine organisms.

Our findings suggest that as atmospheric CO_2_ rises in coming decades, the larvae of *C. virginica*, and perhaps other estuarine species, will grow and calcify more slowly than today. Unless larvae can achieve settling/metamorphic competence more efficiently in the future, slowed growth and calcification will lead to prolonged times in the water column, a situation that can result in higher pre-settlement larval mortality. Mortality from predation and disease increases the longer the larvae remain in the water column [43]–[44]. To illustrate the impact of relatively small changes in water column residence and mortality on recruitment, Kennedy [45] modeled the theoretical fate of *C. virginica* larval offspring from a single female, using natural daily mortality data from a variety of investigators. An 89% reduction in successful recruitment was postulated when metamorphosis was delayed five days (from 20 d to 25 d) and daily mortality rate increased from 20% to 25%. If higher water column mortality rates are coupled with lower larval quality at settlement (i.e., larvae settling with fewer energy reserves), then rates of recruitment could be expected to decrease in the future.

Whether or not prolonged time in the plankton and increased energy expenditures during larval growth and development from elevated CO_2_ adversely affect later life stages and, ultimately, the population dynamics and ecology of *C. virginica* or other species is unknown. Clearly, the larvae of both *Crassostrea* species that we studied have the physiological capacity to grow and calcify in undersaturated conditions, but if and how increasing *p*CO_2_ at all points in their habitat will adversely affect survivorship in the plankton as well as metamorphic success and recruitments dynamics remains to be tested.

On the Pacific coast of the United States, commercial oyster hatcheries are experiencing alarming and widespread difficulties keeping Pacific oyster *(C. gigas)* larvae alive in culture, with two of the largest hatcheries reporting production rates down by as much as 80%. Moreover, there has been little or no “natural” recruitment for several years in areas where *C. gigas* have previously established naturalized populations. (R. Downey, Pacific Coast Shellfish Growers Association, pers. comm.). In regions of upwelling along the continental shelf of western North America, Feely et al. [46] have determined that the pH and Ω_arag_ of surface waters are more acidic and have less aragonite saturation than expected. At water depths of 40–120 m in many locations along the coast, but at 0 m in the region near the California/Oregon border, pH was reported to be ∼7.75 and Ω_arag_<1.0. Whether recent recruitment and aquaculture failures are linked to changes in carbonate chemistry are unknown, but should be investigated.

The natural spatial and temporal heterogeneity in salinity, alkalinity, pH, and *p*CO_2_ in estuaries impose more complex environmental stresses on calcifying biota than in the open ocean where the environment is far less variable. As atmospheric CO_2_ concentrations increase, the proportion of estuarine habitat undersaturated for aragonite will increase. In a high CO_2_ world, we predict that the aragonite compensation point in estuaries (salinity isopleth where Ω_arag_ = 1.0) will shift seaward toward higher salinities. Because estuarine species have evolved in such variable environments, they may possess greater physiological capacity to respond to CO_2_-induced acidification than fully marine taxa. Nevertheless, adaptive physiological tolerance of larvae may not be sufficient to sustain populations of calcifying benthic species, including widespread economically important fisheries, in the face of changing atmospheric CO_2_.
